# Expression and Function of *Ccbe1* in the Chick Early Cardiogenic Regions Are Required for Correct Heart Development

**DOI:** 10.1371/journal.pone.0115481

**Published:** 2014-12-29

**Authors:** João Furtado, Margaret Bento, Elizabeth Correia, José Manuel Inácio, José António Belo

**Affiliations:** 1 Regenerative Medicine Program, Departamento de Ciências Biomédicas e Medicina, Universidade do Algarve, Faro, Portugal; 2 IBB-Institute for Biotechnology and Bioengineering, Centro de Biomedicina Molecular. e Estrutural, Universidade do Algarve, Campus de Gambelas, 8005-135 Faro, Portugal; 3 CEDOC, NOVA Medical School/Faculdade de Ciências Médicas, Universidade Nova de Lisboa, Campo Mártires da Pátria 130, 1169-056 Lisboa, Portugal; Northwestern University, United States of America

## Abstract

During the course of a differential screen to identify transcripts specific for chick heart/hemangioblast precursor cells, we have identified *Ccbe1* (*Collagen and calcium-binding EGF-like domain 1*). While the importance of Ccbe1 for the development of the lymphatic system is now well demonstrated, its role in cardiac formation remained unknown. Here we show by whole-mount *in situ* hybridization analysis that c*Ccbe1* mRNA is initially detected in early cardiac progenitors of the two bilateral cardiogenic fields (HH4), and at later stages on the second heart field (HH9-18). Furthermore, c*Ccbe1* is expressed in multipotent and highly proliferative cardiac progenitors. We characterized the role of c*Ccbe1* during early cardiogenesis by performing functional studies. Upon morpholino-induced c*Ccbe1* knockdown, the chick embryos displayed heart malformations, which include aberrant fusion of the heart fields, leading to incomplete terminal differentiation of the cardiomyocytes. c*Ccbe1* overexpression also resulted in severe heart defects, including *cardia bifida.* Altogether, our data demonstrate that although cardiac progenitors cells are specified in c*Ccbe1* morphants, the migration and proliferation of cardiac precursors cells are impaired, suggesting that cCcbe1 is a key gene during early heart development.

## Introduction

The heart is the first organ to be formed and its circulatory function is critical from early stages for the viability of the developing embryo. In vertebrates, the heart develops from three distinct pools of cardiac progenitors: the cardiogenic mesoderm cells, the proepicardium, and the cardiac neural crest (CNC) cells. These heart cell precursors are positioned in separate embryonic regions, and are subject to distinct molecular signals during development giving rise to the different cardiac structures [1], [2]. In chick, at stage HH4-5, the cardiac precursors are located in the cardiogenic mesoderm (heart forming region) [3]–[5] and are composed by two different populations of heart progenitors, namely the first heart field (FHF) and the second heart field (SHF) [6]. Although, these two distinct mesodermal populations have a common origin, they contribute with different cells types to the developing heart in a temporally and spatially specific fashion [7]. The FHF is derived from the anterior splanchnic mesoderm and contributes to the myocardial cells of the primitive heart tube, which ultimately contributes to the left ventricular region. The SHF lies anterior and dorsal to the linear heart tube and is derived from the pharyngeal mesoderm medial to the heart fields, which will contribute to the outflow tract region [2], [8]. The remaining structures, namely, the right ventricle, the atrioventricular canal and the atria, have contribution from both heart fields [7].

Another pool of cardiac progenitor are the cardiac neural crest cells, which are a subpopulation of the cranial neural crest cells that delaminate from the dorsal neural tube and migrate toward the heart [9]. In chick, the ablation of these cells leads to cardiac outflow tract defects, abnormal myocardial function, and malformations of the derivatives of the caudal pharynx including pharyngeal glans, SHF and arch arteries [9], [10].

The characterization and functional analysis of novel genes involved in cardiogenesis, has major implications for the treatment of congenital and adult heart diseases. Recently, we reported a differential screening in which novel genes required for the development and differentiation of the vertebrate heart and hemangioblast precursor cell lineages were identified [11]. From the 777 detected genes expressed in the heart forming regions (HFR), 199 were classified as upregulated uncharacterized genes [11]. Among these uncharacterized genes was the chick collagen and calcium-binding EGF-like domain 1 (c*Ccbe1*; gene ID: 770043), which predicted amino acid sequence contains a signal peptide for secretion, a collagen and a calcium binding EGF-like domains, highly conserved across vertebrates.

Here, we show that during early chick development c*Ccbe1* is expressed in the early cardiac progenitors that emerge from the primitive streak to form the two bilateral cardiogenic fields at HH4 and in the cardiogenic mesoderm of the FHF and SHF between HH5 to HH8. As development proceeds c*Ccbe1* localizes predominantly in the region of the SHF (HH9 to HH18). In addition, we address the functional role of cCcbe1 in chick early heart development by employing overexpression and knockdown approaches. Through c*Ccbe1* knockdown, the embryos displayed heart abnormalities, the phenotype included aberrant or incomplete fusion of the heart forming regions. Furthermore, c*Ccbe1* morphants embryos demonstrated reduced levels of the proliferation of cells in cardiac regions and in the Hnk1 signal. On the other hand, ectopic expression of c*Ccbe1* resulted in severe heart tube abnormalities, beingwith *cardia bifida* the most common phenotype. Moreover, these embryos demonstrated increased proliferation of cells in cardiac regions and Hnk1 levels in the cardiac neural crest cells and heart tube region. Taken together, these results show that c*Ccbe1* affects the proliferation and the Hnk1 levels of the cardiac progenitors cells, leading to an incorrect development of the heart.

## Materials and Methods

### Ethics Statement

The studies involving animal experiments are in accordance to the ethical issues for clinical research and EU guidelines for animal research. All animal work performed in this study was conducted in compliance with the Portuguese law and approved by the Consultive Commission of the Veterinary Agency from Portuguese Ministry of Agriculture (Directive 2010/63/EU of the European Parliament), the Agency responsible for issuing approval for experimental animal studies, following the EU guidelines for animal research and welfare.

### Morpholinos and DNA constructs

The pCAGGS-GFP vector [12], carrying the cDNA of the green fluorescence protein under the control of the CAGGS promoter, was used to control the extent and efficiency of electroporation.

Chick Ccbe1 overexpression plasmids were based on a modified pCAGGS-MCS-IRES-GFP vector (gift from I. Palmeirin, CBME, UALG). The coding sequence of c*Ccbe1* was amplified by PCR. The c*Ccbe1* coding sequence was isolated by reverse transcriptase (RT)-PCR according to the published sequence (GeneBank accession no. XM_001233357) and subcloned into the *EcoRI* site of pCAGGS-MCS-IRES-GFP.

Fluorescein-tagged antisense morpholinos oligonucleotides cCcbe1MO: 5′-CGCGGCTCTGCGCTCACCTGAAGCA-3′ and CoMO: 5′-CCTCTTACCTCAGTTACAATTTATA-3′ were designed and produced by Gene Tools.

### Chick embryo collection and culture

Fertilized chicken eggs (Sociedade Agrícola Quinta da Freiria, SA, Torres Vedras, Portugal) were incubated for 1–3 days maintained at 38°C in a humidified incubator. Embryos were staged according to Hamburger and Hamilton [13]. For the culture, embryos were explanted at HH3^+^/HH4 together with the vitelline membrane and anchored to a metacrilate ring following the protocol of New [14].

### Early chick embryo electroporation

Embryos were microinjected and electroporated as described previously [15] at HH3^+^/HH4 stage with DNA solution (0.5–3 mg/ml; 0.1% Fast Green; Sigma) in the region fated to form the heart. For the knockdown experiments, control morpholino (CoMO) or cCcbe1 morpholino (cCcbe1 MO) (Gene Tools LLC) were electroporated. For the gain-of-function experiments the control (pCAGGS-IRES-GFP) or cCcbe1 overexpression (pCAGGS-cCcbe1-IRES-GFP) vectors were electroporated. The embryos were then incubated at 38°C for the appropriate period of time (10–30 h), at end of the cultured fixed with 4% paraformaldehyde (PFA) and processed for whole-mount *in situ* hybridization and immunofluorescence. The embryos were observed under a fluorescence stereomicroscope (Leica MZ16FA).

### Whole-mount *in situ* hybridization

Embryos were processed for whole-mount *in situ* hybridization using a standard protocol as previously described [16]. The chick probes were kindly provided by J. Belmonte (*Tbx5)*, A. Munsterberg *(Mhc) and* J. Leon (*Fgf8*). *Islet-1 was obtained from Addagene plasmid 16273.* The chick Ccbe1 (1191-bp) was generated by RT-PCR cloning (clone was obtained from the BBSRC chick EST database: ChEST963b3 for cCcbe1; seq. identifier 603865952F1).

### Immunohistochemistry analyses

Fixed untreated, CoMO, cCcbe1MO, pCAGGS-IRES-GFP (control) and pCAGGS-Ccbe1-IRES-GFP injected embryos were washed with PBS, dehydrated in methanol series and paraffin embedded. Serial 8 µm sections were taken (microtome Leica-RM 2135), dewaxed and rehydrated. After tissue rehydration, antigen retrieval was performed in 10 mM TrisBase/1mM EDTA solution/0.05% Tween20 pH 9.0. Immunostaining was performed using primary antibodies against avian MF20 (1∶200; MF 20-c; DSHB), Phospho-Histone H3 (Ser10) (1∶400; Cell Signaling), HNK1 (1∶200; 1C10; DSHB) and fluorescently coupled secondary antibodies Alexa Fluor 594 goat anti-mouse (1∶800; #A11005; Molecular Probes) or Alexa Fluor 488 goat anti-rabbit (1∶800; #A11008; Molecular Probes). Cell nuclei were labelled with 4′, 6-diamidino-2-phenylindole (1 ug/ml; DAPI; Sigma). Sections were mounted with Mowiol and analysed with Zeiss Axioimager Z2 microscope (Carl Zeiss Group). For quantitation of the Hnk1 signal, fluorescence images of the heart region were processed using ImageJ software. The level of background fluorescence was estimated by averaging background values at four points of each image and was subtracted from the fluorescence. Then areas of fluorescence were marked manually, and fluorescence values were calculated automatically as described elsewhere [17].

### Western blotting

The area pellucida of four cCcbe1 MO injected embryos and respective control MO at stage HH11 embryos was microdissected and suspended in ice-cold lysis buffer consisting of 20 mM HEPES, pH 7.5, 50 mM β-glycerophosphate, 10% glycerol, 2 mM EGTA, 1% Triton X-100, 1 mM sodium vanadate, and the Complete protease inhibitor cocktail (Roche, Indianapolis, IN). The explants were homogenized on ice, centrifuged and transferred to a fresh tube. Upon quantification of the total protein concentrations by the method of Bradford, 10 µg of protein extracts from control and c*Ccbe1* knockdown embryos were loaded on a 12% SDS-PAGE polyacrilamide gel, subjected to electrophoresis and transferred to Hybond-C extra membrane (Amersham Pharmacia Biotech). Blots were probed with the antibody against Ccbe1 (Ccbe1 ab 101967; 1∶500; Abcam, UK) followed by 1 hour at RT with a rabbit polyclonal secondary antibody (Dako, Denmark), developed using a chemiluminescent substrate (Pierce) and analysed using Chemidoc (Bio-Rad).

## Results

### c*Ccbe1* expression during early heart development

**Figure 1:**
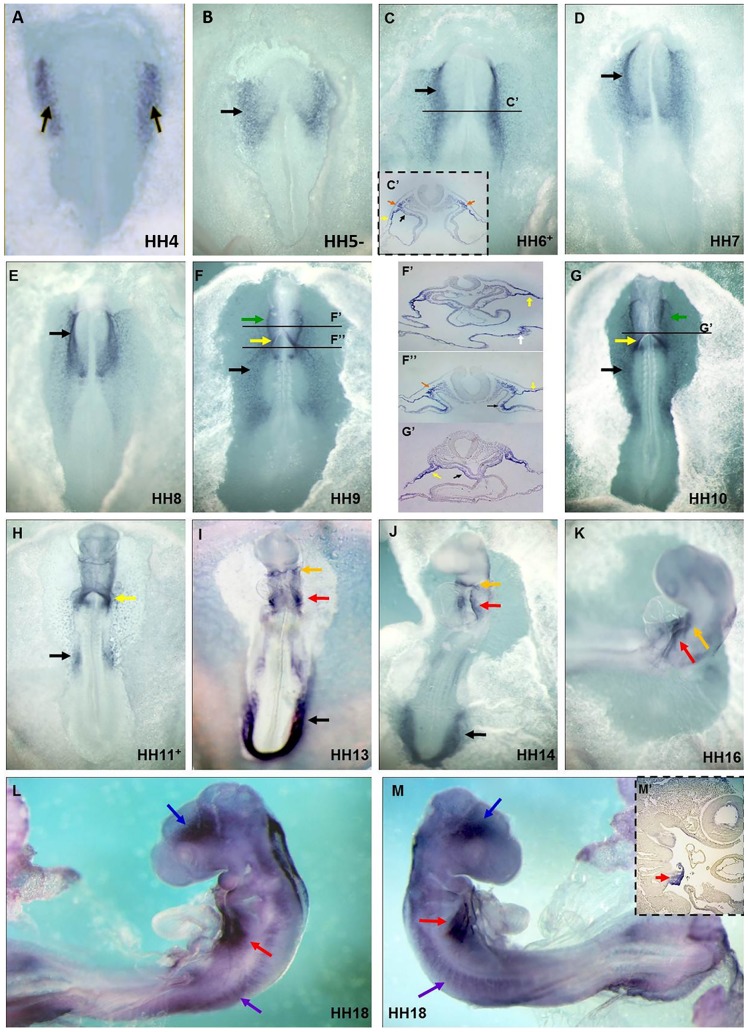
cCcbe1 expression in developing chick embryos. (**A**) Expression of cCcbe1 is present at HH4 in the cardiogenic mesoderm (black arrows); (**B–E**) the expression is present in the anterior lateral plate mesoderm as two patches on either side of the head process (black arrows point to the heart-forming fields); **C**′: Transverse paraffin sections (8 µm) of whole mount stained embryos at stage HH6^+,^ cCcbe1 is expressed in the paraxial mesoderm (orange arrow), splanchnic lateral plate mesoderm (black arrow) and in the somatic lateral plate mesoderm (yellow arrow). (**F–H**) Green arrow point to the lines of expression in the lateral pharynx region; bilateral expression can be observed in the sino-atrial region (yellow arrow), and in the lateral plate mesoderm (black arrow); (**I–K**) Expression can be seen in the tail bud (black arrow); in a specific region behind the heart, known as the SHF (red arrow) and near the anterior part of the heart (orange arrow, conus arteriosus region); **F**′**–F**′′ and **G**′: Transverse paraffin sections (8 µm) of whole mount stained embryos at stage HH9 (F) and HH10 (G) respectively (from anterior to posterior). The sections are oriented with dorsal up and ventral down. (**F**′) Expression of cCcbe1 can be observed at the level of the somatic lateral plate mesoderm (yellow arrow) and also some expression is labelled in the endoderm (white arrow); (**F**′′) Somatic and splanchnic lateral plate mesoderm (yellow arrow and black arrow) are labelled with cCcbe1, and continues partially into the paraxial mesoderm (orange arrow); (**G**′) cCcbe1 expression is seen in the somatic lateral plate mesoderm (yellow arrow) and in the splanchnic mesoderm of the ventral pharyngeal mesoderm (black arrow); (**L–M**) Lateral view of the embryo with expression above de eye (blue arrow), in the SHF region (red arrow) and also some expression is seen around the somites (purple arrow); **M**′: Sagittal paraffin section (8 µm) of whole mount stained embryos at stage HH18, cCcbe1 is expressed in the region of the SHF (red arrow). All embryos are ventral side up and anterior to the top except for J–M that are lateral views.

**Figure 2:**
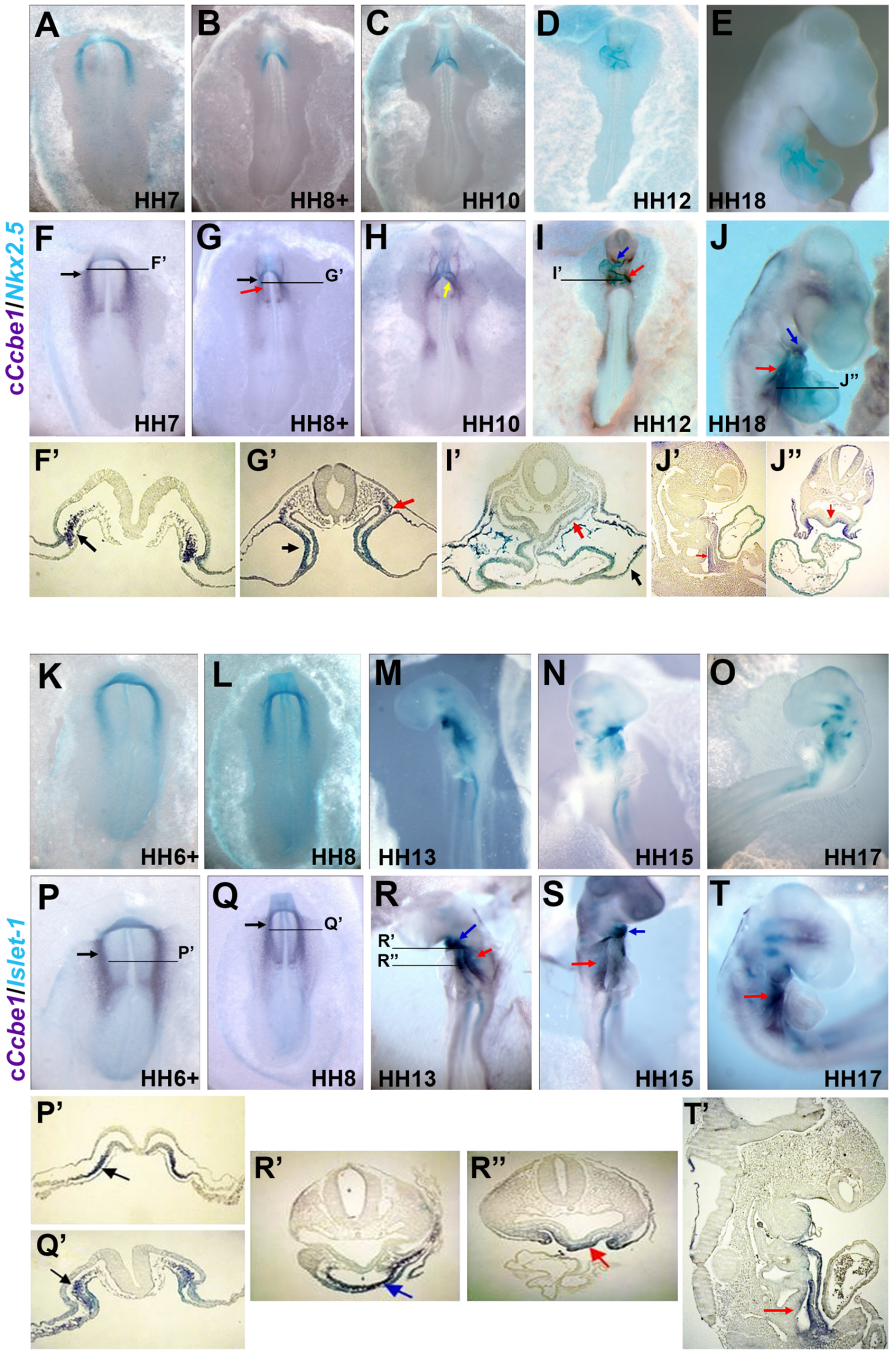
Double WISH analysis of cCcbe1 and the cardiac makers Nkx2.5 and Islet-1. (**A–T**) Comparative expression of c*Ccbe1*, *Nkx2.5* and *Islet-1* during early heart development. All are ventral views except for E, J, M, N, O, R, S and T that are lateral views (anterior to top). (A–E) *In situ* hybridization for *Nkx2.5.* (**F–J**) Double *in situ* hybridization for c*Ccbe1* and *Nkx2.5* (HH7-18); c*Ccbe1* and *Nkx2.5* have overlapping patterns of expression in the heart fields (F and G; black arrow) and in the sino-venosus (H; yellow arrow); **F**′**:** Transverse paraffin sections (8 µm) of double stained embryos at stage HH7, c*Ccbe1* and *Nkx2.5* are co-expressed in the cardiogenic mesoderm of the heart forming fields (black arrow); **G**′ Transverse paraffin sections (8 µm) of double stained embryos at stage HH8^+^, c*Ccbe1* and *Nkx2.5* are co-labeled in the ventrolateral aspect of the splanchnic mesoderm (black arrow) and in the dorsomedially region of the splanchnic mesoderm (red arrow); (**I**) Co-expression in the region of the conus arteriosus (blue arrow) and in the ventral pharyngeal mesoderm (red arrow, SHF); **I**′**:** Transverse paraffin sections (8 µm) shows, an overlapping expression in the endoderm (black arrow), in the region of the conus arteriosus (blue arrow) and a pale co-expression in the splanchnic mesoderm (SHF; red arrow); (**J**) c*Ccbe1* and *Nkx2.5* have overlapping patterns of expression in the SHF (red arrow) and in the conus arteriosus region (blue arrow); **J**′**–J**′′**:** Sagittal (J′) and transverse (J′′) paraffin sections (8 µm) at stage HH18 shows c*Ccbe1* and *Nkx2.5* co-expressed in the region of the SHF (red arrow). (**K–O**) *In situ* hybridization for *Islet-1.* (**P–T**) Double *in situ* hybridization for c*Ccbe1* and *Islet-1;* (**P–Q**) c*Ccbe1* and *Islet-1* have overlapping patterns of expression in the anterior lateral plate mesoderm (black arrows); **P**′**:** Transverse paraffin sections (8 µm) of double stained embryos at stage HH6^+^, shows an overlapping expression in the cardiogenic mesoderm of the heart forming fields (black arrow); **Q**′**:** Transverse sections of double stained embryos at stage HH8, both are co-labeled in the dorsomedially region of the splanchnic mesoderm (black arrow). (**P–T**) co-expression of c*Ccbe1* and *Islet-1* is observed in the caudal part of the distal outflow tract of the heart (conus arteriosus, blue arrow) and in the ventral pharyngeal mesoderm (SHF; red arrow); **R**′**–R**′′**:** Transverse sections shows, a co-expression in the region of the conus arteriosus (R′; blue arrow) and in the splanchnic mesoderm of the SHF (R′′; red arrow); **T**′**:** Sagittal paraffin section (8 µm) of double stained embryos at stage HH18, c*Ccbe1* and *Islet-1* are co-expressed in the region of the SHF (red arrow).

Whole-mount *in situ* hybridization (WISH) revealed that c*Ccbe1* mRNA is first detected at stage HH4 as two patches on each side of the primitive streak, in the regions that correspond to the bilateral cardiogenic mesoderm, the so-called heart forming regions (HFR) (Fig. 1A, black arrow). This expression is maintained until the fusion of the HFR into a straight heart tube at stage HH9 (Fig. 1B–F; black arrow). To determine whether c*Ccbe1* expression can be correlated with the first, second or both heart field populations, double WISH for c*Ccbe1* and the cardiac markers *Nkx2.5* (FHF and SHF) and *Islet-1* (SHF), were carried out. Our results showed that these genes have overlapping expression patterns in the heart fields (Fig. 2A–B and 2F–G). We note that *Nkx2.5* and *Islet-1* only starts to be expressed at HH5^+^-6, whereas c*Ccbe1* expression begins earlier by HH4. A more detailed analysis, throughout transverse sections of whole mount stained embryos at stage HH6^+^/HH7, revealed that c*Ccbe1* is expressed in the paraxial mesoderm (orange arrows), somatic mesoderm (yellow arrow) and in the splanchnic mesoderm (black arrows) (Fig. 1C′). In fact, the expression of c*Ccbe1* is co-labelled with *Nkx2.5* and *Islet-1* throughout the splanchnic mesoderm where the cardiac precursor cells of the FHF and SHF have been identified (Fig. 2A′′–B′′ and 2F′′–G′′, respectively). From stage HH9 to HH11, c*Ccbe1* transcripts were detected near the posterior part of the heart (venous pole), namely, in the sino-atrial region (Fig. 1F–H, yellow arrow). More cranially, c*Ccbe1* expression was observed in the pericardial region: in the endoderm, in the splanchnic, somatic and paraxial mesoderm, and in the mesoderm lateral to the pharynx (Fig. 1F–G′, white, black, yellow, orange and green arrows; respectively). From stage HH13 to stage HH18, the expression of c*Ccbe1* was found in the mesoderm that surrounds the pharynx (pharyngeal mesoderm, SHF), which, at this time, contributes with cells for heart tube elongation (Fig. 1I–M′ red arrow). Double WISH analysis revealed that c*Ccbe1* is co-expressed with *Nkx2.5* and *Islet-1* in this region, indicating that c*Ccbe1* is indeed expressed in the SHF (Fig. 2I–J′′, red arrow; 2R–T′, red arrow; respectively). In addition, c*Ccbe1* and *Nkx2.5* expression co-localize in the *conus arteriosus* (Fig. 2I–J, blue arrow). Furthermore, c*Ccbe1* was found in the caudal part of the distal outflow tract of the heart tube, overlapping *Islet-1* expression in the SHF region (Fig. 2R–R′, blue arrow), Taken together, our data demonstrates that the expression of c*Ccbe1* during avian heart development is initially detected in the FHF and SHF and later is highly specific of the SHF region.

### c*Ccbe1* knockdown leads to aberrant heart formation

**Figure 3:**
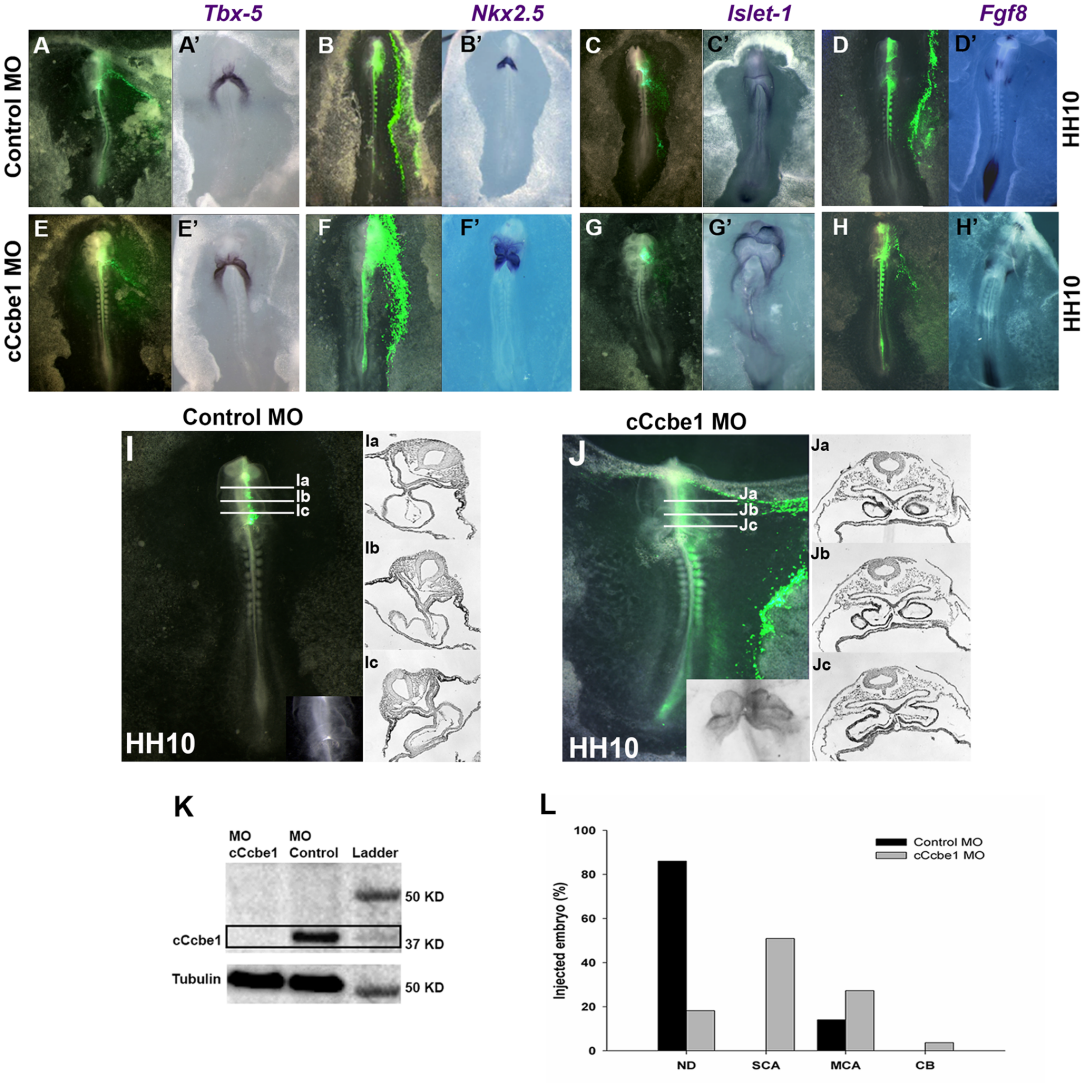
cCcbe1 loss-of-function leads to heart malformations. The embryos were first targeted at stage HH3^+^/HH4 with the designed morpholino and were collected later in development between stage HH9^+^ and HH10. (**A–D**′′) Embryos injected with the control morpholino. (**E–H**′) Embryos injected with the cCcbe1 MO. (**A–H**) Localization and efficiency of the MO injection by detection of fluorescein expression. (**A**′**–**H′**)** Detection of *Tbx5*, *Nkx2.5*, *Islet-1* and *Fgf8* expression by WISH of the injected embryos with CoMO and cCcbe1 MO, respectively. (**I–J**) Histological analysis of control and Ccbe1 Knockdown embryos. (Ia-Jc) Paraffin transverse sections through the heart of the targeted embryos at HH10; fusion of the heart and foregut at the ventral midline is highly abnormal in Ccbe1 Knockdown embryos. All embryos are ventral, except D that is dorsal side up. (K) Western blot analysis of the cCcbe1 MO and Control MO embryos. It was possible to see a loss in cCcbe1 protein levels observed in the cCcbe1 knockdown in comparison to morpholino control embryos. (**L**) Analysis of the phenotypes caused by electroporated embryos with cCcbe1 MO and Control MO. Bar charts showing the percentage of chick embryos presenting cardiac alterations after injection with Control or cCcbe1 MO. Only embryos at stage HH9 and later were considered to this analysis. The total of samples analyzed (n): 100 Control MO and 110 cCcbe1 MO embryos. The y-axis represents the percentage of embryos. The x-axis represents the defects: normal development (ND), severe cardiac alterations (SCA), moderate cardiac alterations (MCA) and cardia bifida (CB).

To dissect the role of c*Ccbe1* during early chick heart development, we used a splicing inhibitory morpholino oligonucleotide to knockdown c*Ccbe1* (cCcbe1MO). Using a standard morpholino as control (CoMO), we injected embryos at HH3^+^ with each morpholino (1 mM), either on the left or/and the right side of the primitive streak, followed by *in* *vivo* electroporation. Both morpholinos were fluorescein tagged at the 3′ end, which enabled us to assess injection efficiency and stability of the morpholino over time. Interestingly, no differences in the phenotype or in the percentage of defects were observed between each side (data not shown). Furthermore, the reduction of Ccbe1 protein in embryos injected with cCcbe1MO was confirmed by Western blot analysis when compared with CoMO injected embryos (Fig. 3K).

During normal early heart development, the bilateral heart fields are drawn towards the midline and fuse into a single heart tube by HH9. How these cardiac precursor cell move back towards the midline is still not clear. In contrast, large numbers of the Ccbe1MO injected embryos fail to form the heart tube. This disrupted development of the heart is well visualized in Fig. 3E–H′ and J–Jc. The knockdown experiments showed that 81.8% of the cCcbe1MO injected embryos displayed significant cardiac malformations when compared with the CoMO injected embryos (Fig. 3L). We classified these cardiac alterations in three classes: severe (50.9%), when the bilateral heart fields are capable to migrate towards the ventral midline, but fail to fuse and form single heart tube (Fig. 3J); moderate phenotype (27.2%), encompassing embryos with a nearly normal heart tube, although not well assembled; and *cardia bifida,* when we have the presence of two totally separated “hearts” on each side of the primitive streak (3.7%). Only embryos at stage HH9 and older were considered for these plots, since earlier than stage HH9 is impossible to classify the heart defects just by observation, once the fusion of the heart tubes only occur at HH9. To better analyse the *cCcbe1* knockdown phenotype, the hearts were sectioned transversaly, and several types of defects were observed. In control embryos, we observed a single heart tube assembled in the ventral midline (Fig. 3I-Ic). In contrast, in the knockdown embryos the bilateral heart fields fail to fuse properly at the midline (Fig. 3J-Jc).

Moreover, to better understand the role of *cCcbe1* during early heart development, gene expression analysis was performed throughout whole-mount *in situ* hybridization of well-characterized cardiogenic markers *Tbx-5* (Fig. 3E′), *Nkx2.5* (Fig. 3F′), *Islet-1* (Fig. 3G′), and *Fgf8* (Fig. 3H′). These genes were chosen because they are expressed in some of the regions where *cCcbe1* is expressed and have a role during early cardiogenesis, more specifically in the SHF.

This data revealed that, even though c*Ccbe1* knockdown causes severe heart dystrophy, the temporal and spatial expression of these markers seems not to be altered in c*Ccbe1* knockdown embryos up to stage HH11. This suggests that c*Ccbe1* might not be required for the specification and determination of the heart fields, but instead for the morphogenetic patterning of the cardiogenic mesoderm.

**Figure 4:**
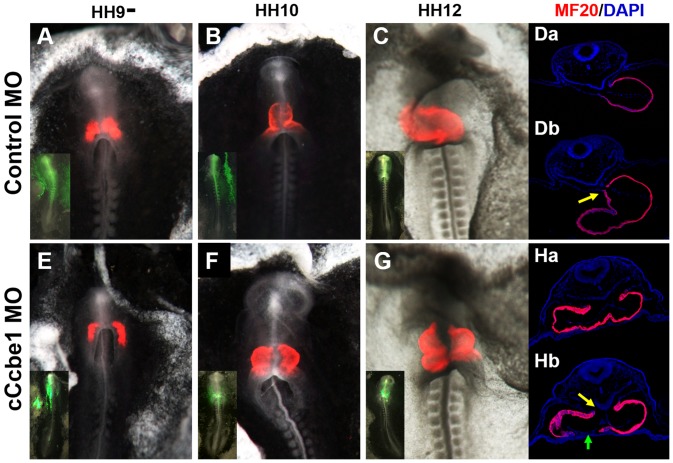
Immunofluorescence analysis of cCcbe1 and Control MO in chick embryos. Embryos were target at HH3+/HH4 with cCcbe1 (cCcbe1 MO) and Control (CoMO) morpholino and allowed to develop until stage HH12. Some embryos were subsequently analyzed by whole mount (A–C; E–G) or in sections (Da, Db, Ha and Hb) immunofluorescence staining for MF20 (myocardium: red; Dapi: blue). (A–C) Embryos injected with CoMO showed no cardiac malformations. (E–G) embryos injected with cCcbe1 MO showed alterations in cardiac tube fusion: a delay in the fusion (E), fusion failure (F) and incomplete fusion (G). (Da-Db) Transverse sections (8 µm) of embryos electroporated with CoMO at the level of the heart. (Ha-Hb) Transverse sections (8 µm) of embryos electroporated with cCcbe1 MO at the level of the heart: these images highlight the malformations in the closure of the dorsal mesocardium (yellow arrowhead) and the lack of cell expressing myosin heavy chain (green arrowhead) caused by a failure on initiation of cardiac differentiation at the ventral midline.

Next, we performed immunofluorescence staining with sarcomeric myosin heavy chain (MF20), in whole mount and in sections, and follow the fusion of bi-lateral cardiac fields upon the formation of the heart tube. MF20 is a marker for terminally differentiated cardiomyocytes. At stage HH9^−^, we observed that the heart fields in the cCcbe1Mo injected embryos were further appart than the control embryos (Fig. 4A and E). This suggests that the fusion of the heart fields are somewhat delayed in the absence of c*Ccbe1*. Later, at stage HH10-12, the two heart fields fail to fuse properly at the ventral midline, exhibiting a gap in the MF20-positive cells between them (Fig. 4F–G). Likewise, the same defect was observed in transverse sections, with the presence of cells in the heart, that do not express MF20, (Fig. 4Hb, green arrow) sugesting that the embryos fail to undergo terminal differentiation at the midline. n addition, the closure of the dorsal mesocardium seems to be also affected (Fig. 4 Ha-Hb, yellow arrow). The dorsal mesocardium is a transient structure formed when the splanchnic mesoderm (SHF) of opposite sides of the embryo come together from dorsal and ventral to the heart, forming double layered supporting membranes. After the rupture of the dorsal mesocardium the heart tube closes dorsally and the dorsal pericardial walls fuse, something that in the c*Ccbe1* morphant embryos seems also to fail. Taken together, these data indicate that the bi-lateral fields fail to fuse properly at the midline in the absence of c*Ccbe1,* leading to the development of an aberrant heart tube. The results suggest that this phenotype is not due neither to specification nor determination of the cardiac precursors, since the expression of *Nkx2.5* seems to be normal, but to a failure in terminal differentiation.

### c*Ccbe1* overexpression leads to *cardia bifida*

**Figure 5:**
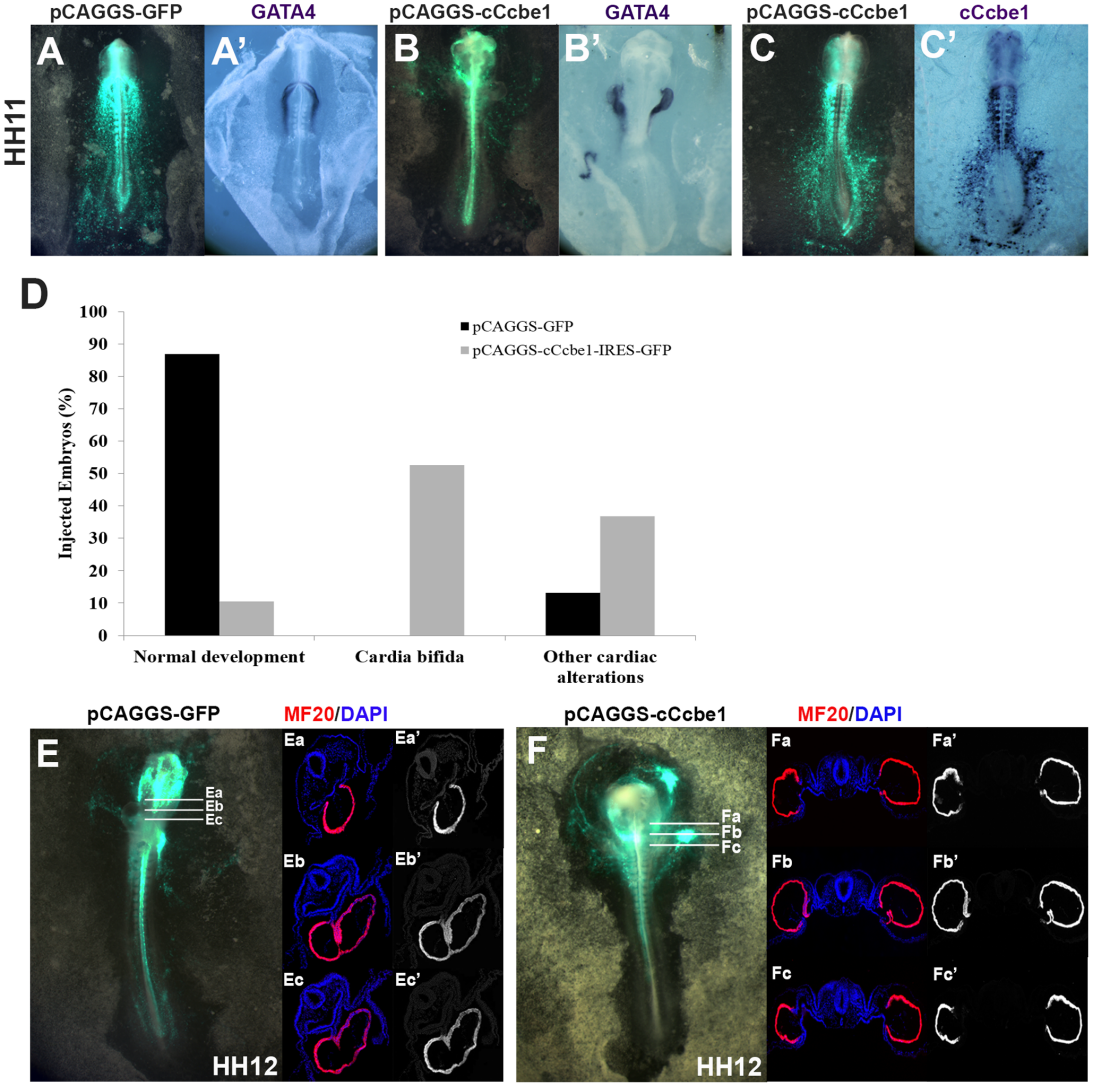
cCcbe1 gain-of-function in chick embryos. Embryos were targeted at stage HH3^+^/HH4 with the control vector pCAGGS-IRES-GFP (A) or with the overexpression vector pCAGGS-cCcbe1-IRES-GFP (B–C) and collected at stage HH11. (**A**) Localization and efficiency of the control vector pCAGGS-GFP injection by detection of fluorescein expression. (**A**′) Embryos injected with control vector showed no cardiac malformations detected with *Gata4* by WISH. (**B–C**) Localization and efficiency of the overexpression vector pCAGGS-cCcbe1-IRES-GFP injection by detection of fluorescein expression. (**B**′) Embryos injected with pCAGGS-cCcbe1-IRES-GFP showed alterations in cardiac tubes fusion detected with *Gata4* by WISH, namely, bifid heart was observed (formation of two separate heart tubes). (**C**′**)** Detection of c*Ccbe1* expression by WISH, demonstrating that pCAGGS-cCcbe1-IRES-GFP is overexpressing c*Ccbe1*. (**D**) Analysis of the defects caused by electroporated embryos with control vector pCAGGS-IRES-GFP or overexpression vector pCAGGS-cCcbe1-IRES-GFP. Bar charts showing the percentage of chick embryos presenting cardiac alterations after injection with control vector or overexpression vector. Only embryos at stage HH9 and later were considered to this analysis. The total of samples analyzed (n): 38 control vector and 38 overexpression vector embryos. The y-axis represents the percentage of embryos. The x-axis represents the defects: normal development, cardia bifida and other cardiac alterations. (**E–F**) Some embryos were subsequently analyzed immunohistochemistry staining for MF20 (myocardium: red; Dapi: blue) in transverse sections (8 µm). (**E–Ec**′) Embryos injected with control vector showed no cardiac malformations. (**F–Fc**′) embryos injected with overexpression vector showed *cardia bifida* defects. These images showed none alteration of cell expressing MF20.

We then used a gain-of-function approach to overexpress c*Ccbe1*. Therefore, we designed a vector with the c*Ccbe1* under the control of the constitutive promoter CAGGS (pCAGGS-cCcbe1-IRES-GFP), and the backbone vector, pCAGGS-IRES-GFP, to be used as a control. Embryos at HH3^+^/HH4 were injected with each vector on the right and left sides of the primitive streak, followed by *in* *vivo* electroporation, and a WISH was performed to confirm the overexpression of c*Ccbe1*. Indeed, injection of pCAGGS-cCcbe1-IRES-GFP resulted in a co-localized c*Ccbe1* expression with GFP, including regions where endogenous c*Ccbe1* is not detected (Fig. 5C–C′).

According to our data, after 18 h to 24 h of incubation the injected embryos with pCAGGS-cCcbe1-IRES-GFP displayed severe heart tube malformations (Fig. 5B–B′), absent in control vector-injected embryos (Fig. 5A–A′). A detailed analysis in the distribution of the phenotypes showed that the heart of almost all the control vector injected embryos developed normally (86.8%) (Fig. 5D). The remaining 13.2% presented mild cardiac alterations, but none of them displayed *cardia bifida*. In contrast, 89.5% of the c*Ccbe1* overexpressed embryos showed significant cardiac alterations (Fig. 5D). Among these, 52.6% displayed *cardia bifida* and 36.9% milder cardiac alterations, i.e. the heart fields were able to migrate to the midline but fail to fuse properly, and the hearts consistently failed to undergo looping of the heart tube (Fig. 5D).

Moreover, MF20 immunofluorescence analysis showed that the cardiac fields of c*Ccbe1* overexpressed embryos remain close to the lateral plate mesoderm and consequently the embryos showed a bifid heart phenotype (Fig. 5Fa–Fc′). This failure on the migration of the cardiac fields towards the midline is strikingly similar to those observed in embryos lacking the cardiac transcription factor, *Gata4*. In these embryos, two separate heart tubes develop in the majority of mutants [18], [19]. Due to the similarities between the phenotypes of embryos in which c*Ccbe1* is overexpressed and null-mutants for *Gata4*, we examined the expression pattern of *Gata4* in embryos injected with pCAGGS-cCcbe1-IRES-GFP. The results showed that, despite the development of *cardia bifida* in the c*Ccbe1* overexpressed embryos it is unlikely that the overexpression of this gene interfere with the expression of *Gata4* up to stage HH11 (Fig. 5A–B′).

### c*Ccbe1* affects the proliferation of the cardiac cells

Cell proliferation, while not the only mechanism, is an important process for the formation of the heart [20], [21]. Nevertheless, it is known that although the initially formed myocardial tube continues to grow, the newly formed heart tube is a non-proliferating structure, implying that its growth occurs by recruitment of cells from the flanking mesoderm, more specifically the splanchnic and pharyngeal mesoderm [22]–[24]. Usually, if something interfers with the ability of these cells to replicate at this stage heart defects will became apparent.

**Figure 6:**
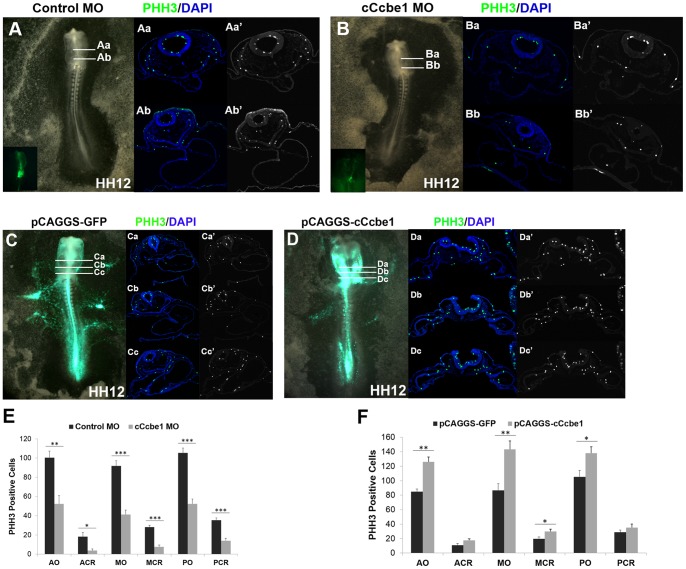
cCcbe1 loss and gain of function disturbs cell proliferation in chick embryos. (**A–B;E**) cCcbe1 loss of function; Embryos at stage HH3^+^/HH4 were target with the cCcbe1 MO (B) and CoMO (A), and developed until HH12. Embryos were transverse sectioned and then immunohistochemistry staining was performed for PHH3 (green; Aa–Bb′). (**A–Ab**′) Control morpholino treated embryos showing normal heart development and proliferation; (**B–Bb**′) cCcbe1 treated embryos showing heart alterations and a decrease in proliferating cells. Note that at this stage the heart is not proliferative, therefore the region of the pharyngeal and splanchnic mesoderm was taken in consideration (SHF contribution). Dapi: blue; PHH3: green. (**E**) Analysis of the cCcbe1 knockdown in cardiac cells proliferation. (**C–D; F**) cCcbe1 gain of function; Embryos at stage HH3^+^/HH4 were target with the control vector pCAGGS-IRES-GFP (C) or with the overexpression vector pCAGGS-cCcbe1-IRES-GFP (D) and developed until HH12. Embryos were transverse sectioned and then immunohistochemistry staining was performed for PHH3 (green; Ca–Dc′). (**C–Cc**′) Control treated embryos shows normal heart development and proliferation. (**D–Db**′) Overexpression-cCcbe1 treated embryos shows heart alterations and an increase in proliferating cells. Dapi: blue; PHH3: green.; (**F**) Analysis of the cCcbe1 overexpression in cardiac cells proliferation. Embryos were subsequently analyzed in transverse sections by immunohistochemistry staining for PHH3. Proliferating cells were counted in 2 distinct regions: cardiac region (pharyngeal and splanchnic mesoderm) and overall (all the regions in the embryo: cardiac and non-cardiac). The total of embryos analyzed (n): 4 control MO, 4 pCAGGS-cCcbe1, 4 cCcbe1 MO and 4 pCAGGS-cCcbe1. The y-axis represents the PHH3 positive cells. The x-axis represents the regions of the counted PHH3 positive cells: anterior overall (AO), anterior cardiac region (ACR), medial overall (MO), medial cardiac region (MCR), posterior overall (PO) and posterior cardiac region (PCR). Error bars represent the S.E.M. from four replicates. *p<0.05; **p<0.01; ***p<0.001.

To test whether c*Ccbe1* is involved in the proliferation of heart precursor cells, we analysed cell proliferation through immunostaining for phospho-Histone H3 (PHH3), a marker of mitotic cells, on transverse sections of CCBE1 knockdown and control embryos (Fig. 6A–B). These data revealed a significant reduction in the number of PHH3-positive cells in c*Ccbe1* morphant embryos (Fig. 6Aa, Ba). To better understand the role of cCcbe1 in the proliferation of cardiac precursor cells, we quantify cell proliferation in the cardiac region (splanchnic and pharyngeal mesoderm regions) and in the overall embryo (cardiac plus non-cardiac regions). Moreover, we selected sections at the anterior, medial and posterior levels of the heart tube region and count the number of proliferating cells. The results showed that cCcbe1MO embryos presented an overall decrease (ratio 2∶1) of proliferating cells when compared with the control MO embryos and that in the cardiac cells this difference in proliferation was even higher (ratio 3∶1) (Fig. 6E).

These results indicate that the absence of cCcbe1 in the chick embryo decreased cell proliferation in both pharyngeal and splanchnic mesoderm where cCcbe1 is normally expressed (Fig. 1G′′, black arrow), suggesting a role of cCcbe1 for proper proliferation of the SHF progenitors. When cell proliferation was examined in c*Ccbe1* overexpressed embryos, as expected, an increase in the number of the proliferating cells was observed in the cardiac region (Fig. 6C–D). Nevertheless, this cell proliferation increase was also observed in other regions of *cCcbe1* gain-of-function embryos (Fig. 6F).

### c*Ccbe1* loss and gain-of-function affects Hnk1 expression

**Figure 7:**
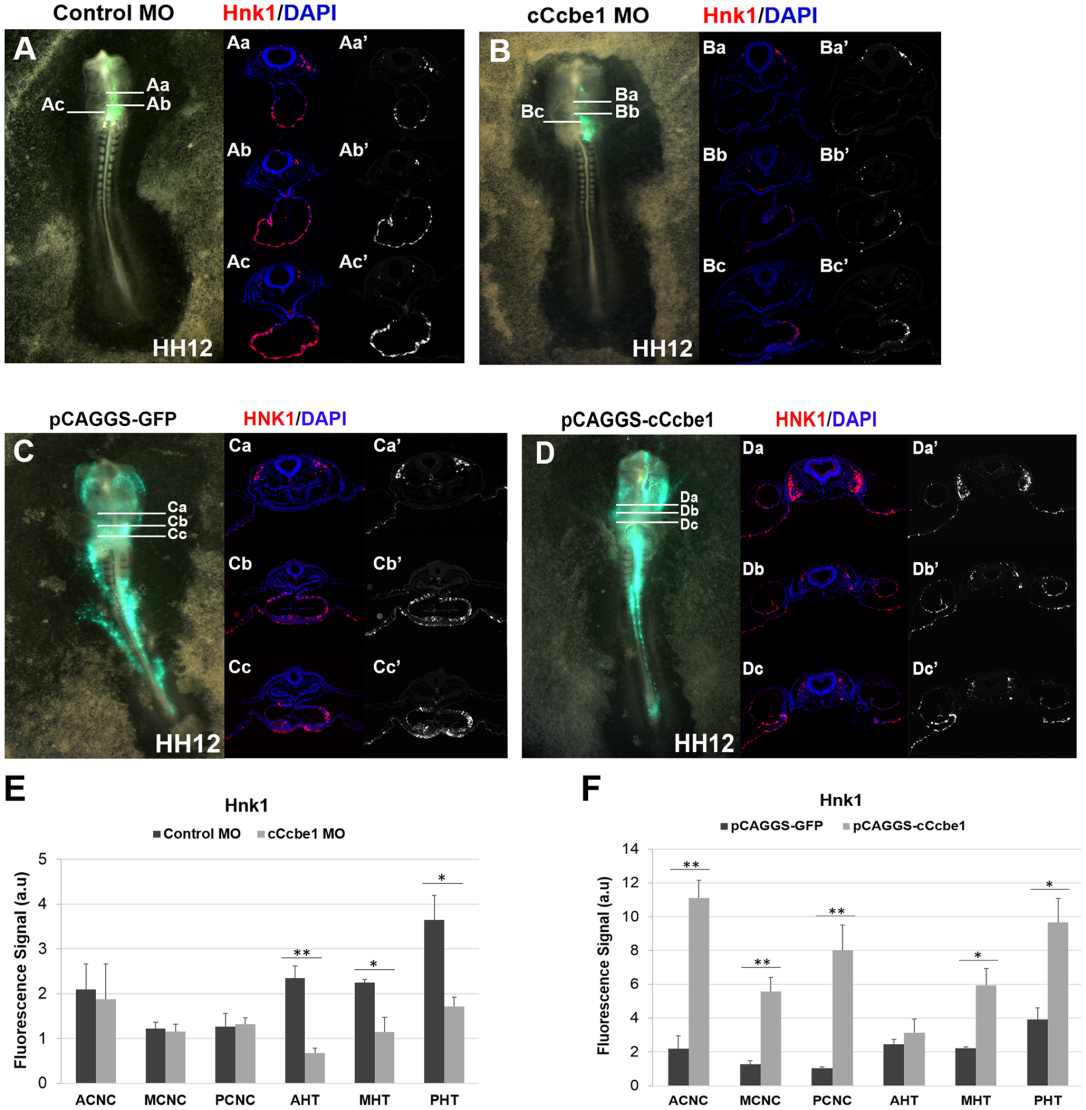
Hnk1 immunofluorescence analysis of cCcbe1 loss and gain of function in chick embryos. (A–B; E) cCcbe1 loss of function; Embryos were target at HH3^+^/HH4 with cCcbe1 and Control morpholino and allowed to develop until stage HH12 (A–B). Embryos were subsequently analyzed transversal sections (Aa-Bc) by immunostaining for Hnk1 (Hnk1: red; Dapi: blue); (Aa–Ac′) Transverse sections (8 µm) of embryos electroporated with CoMO at the level of the heart; Hnk1 expression is detected through the heart tube and CNC. (Ba–Bc′) Transverse sections (8 µm) of embryos electroporated with cCcbe1 MO at the level of the heart: these images highlight the lack of Hnk1 expression in the heart tube. (E) Quantitative analysis of Hnk1 immunostaining in two distinct regions: cardiac neural crest (CNC) cells and heart tube (HT). (C–D; F) cCcbe1 gain of function; Embryos were target at HH3^+^/HH4 with the overexpression vector pCAGGS-cCcbe1 and the control vector pCAGGS-GFP and allowed to develop until stage HH12 (C–D). Embryos were subsequently analyzed transversal sections (Ca-Cc) by immunostaining for Hnk1 (migrating CNC and conducting system marker: red; Dapi: blue). (Ca–Cc′′) Transverse sections (8 µm) of embryos electroporated with control vector at the level of the heart; Hnk1 expression is detected through the heart tube and CNC. (Da–Dc′′) Transverse sections (8 µm) of embryos electroporated with overexpression vector at the level of the heart: these images highlight increase of Hnk1 expression in the heart tube and CNC. (F) Quantitative analysis of Hnk1 immunostaining in two distinct regions: cardiac neural crest (CNC) cells and heart tube (HT). The total of embryos analyzed (n): 3 control MO, 3 pCAGGS-cCcbe1, 3 cCcbe1 MO and 3 pCAGGS-cCcbe1. The y-axis represents the Hnk1 fluorescence signal. The x-axis represents the regions of the Hnk1 measured signal: anterior CNC (ACNC), medial CNC (MCNC), posterior CNC (PCNC), anterior heart tube (AHT), medial heart tube (MHT), posterior heart tube (PHT). Error bars represent the S.E.M. from three replicates. *p<0.05, **p<0.01.

Hnk1 is a glycoprotein known to play an active role in the migration of neural crest cells [25], [26], and expressed in the cells of the SHF as they move into the outflow tract [22]. To determine if c*Ccbe1* loss- and gain-of-function influences Hnk1 expression in the chick embryo, we performed immunostaining with the Hnk1 antibody on transverse sections at the heart region of c*Ccbe1* knockdown, overexpression and respective control embryos (stage HH12) (Fig. 7A–B and 7C–D). The results showed that the level of Hnk1 signal was decreased in c*Ccbe1* morphants on sections at the anterior, medial and posterior levels of the heart tube region when compared to the same regions in the control embryos (Fig. 7Aa–Bc′ and E). Nevertheless, while this decrease in the level of Hnk1 signal was consistent in the heart of all analysed embryos, there was no obvious difference in most of the embryos at the level of the CNC cells. On the other hand, when comparing control and c*Ccbe1* overexpressed embryos, the Hnk1 signal was increased in both CNC cells and heart tube regions in the c*Ccbe1* overexpressed embryos (Fig. 7F). Moreover, the intensity of Hnk1 staining in the CNC cell seems to be wider and stronger in the c*Ccbe1* overexpressed embryos when compared to the CNC cells on the control embryos. Taken together, these results suggest that altered levels of cCcbe1 caused by gain and loss of function analysis affect the migration of CNC cells.

## Discussion

The importance of *Ccbe1* for the development of the lymphatic system is indisputable [27], [28]. However, its role in cardiac development remained unknown. Nevertheless, several lines of evidence emphasize the role of Ccbe1 in early heart development. First, in humans, while mutations in *CCBE1* are associated with Hennekam syndrome, a disorder characterized by abnormal lymphatic system development, some of these patients present as well congenital heart defects [29], [30]. Second, the expression analysis has shown that m*Ccbe1* is expressed in heart precursors of the FHF, SHF and proepicardium in mouse embryos from embryonic day (E)7.0 to E9.5 [31]. Furthermore, analyses of m*Ccbe1* heterozygous knockout embryos have shown X-Gal staining at the mesothelium of the heart at E12.5 [28].

Here, we describe for the first time the role of *cCcbe1* in early chick heart development.

Expression analysis showed that c*Ccbe1* is detected at stage HH4 on either side of the primitive streak where the cells with cardiogenic potential are located. As the cells migrate to the anterior lateral plate mesoderm, they became committed to a cardiac fate resulting in the formation of the first and second heart fields [6]. *Ccbe1* is strongly expressed in the splanchnic mesoderm as this germ layer splits. Later at HH12-18, its expression is restricted to the SHF, in the region where the undifferentiated cells proliferate continuously and migrate to populate the heart tube. This expression pattern, is very similar to the one described for *Islet-1,* which is expressed in cells that display rapid proliferation but ceases upon cell differentiation [23], [32]. Likewise, the evident expression of c*Ccbe1* in the cardiogenic mesoderm and in the primordial myocardial tissues is not detected at later stages of heart development. This suggests that c*Ccbe1* expression is limited to multipotent and highly proliferative progenitors but downregulated after cardiac commitment. Interestingly, the same pattern of expression was observed in mice, where *Ccbe1* expression was detected in the major populations of cardiac progenitors, namely the FHF, SHF and proepicardium [31].

The c*Ccbe1* functional studies presented here clearly suggest that *Ccbe1* has a role in early cardiac development. The downregulation of cCcbe1 at stage HH4, as the cells are reaching the heart fields from the primitive streak, results later (stage HH10) in an incorrect formation of the heart tube, i.e., the bilateral cardiac progenitors are capable to migrate back towards the ventral midline but the process of fusion locally fall through. On the other hand, the overexpression of cCcbe1 leads to a *cardia bifida* in which the cCcbe1 expressing cells in the first heart field do not migrate towards the midline, resulting in formation of two heart-like structures on each side of the lateral plate mesoderm. Interestingly, both knockdown and overexpression functional studies suggest that cCcbe1 do not affect cardiomyocytes commitment. The embryos targeted with cCcbe1 MO, continuously expressed *Nkx2.5, Tbx5, Fgf8 and Isl1* specification markers, while the ectopic expression of *cCcbe1* cannot induce a consequent activation of Gata4 expression, the transcriptional activation of this cardiogenic marker remained unaltered. As mentioned earlier, the heart precursor cells proliferate and migrate as they travel towards their final destination at the midline to form the heart primordia and where they differentiate into cardiomyocytes [7]. In the chick this happens by stage HH9 of development at the beginning of the fusion of the heart tube [33], [34]. According to our data, the cells at the ventral midline in c*Ccbe1* knockdown embryos do not express the cardiomyocytes marker MF20, suggesting that these cells fail to initiate cardiac terminal differentiation. Therefore, c*Ccbe1* is not important for cardiomyocyte commitment but likely has a role in the proper proliferation and migration of the cardiac precursor cells to form the heart tube. Curiously, in Ccbe1 knock-out mice, during lymphangiogenesis the lymphatic progenitors are also specified, but they are unable to migrate away from the cardinal vein [28].

The morphogenetic movements involved in heart tube formation required several cytoskeletal, adhesive, and extracellular structural proteins and their regulators. Several proteins of the extracellular matrix such as laminin, tenascin and fibronectin have been implicated in early heart development [35]–[38]. These molecules, similarly to Ccbe1, also contain EGF-like domains and it has been suggested that EGF-like domains in ECM protein signals for cellular growth and differentiation [39].

For example, the deposition of fibronectin at the ventral midline is required for the movement of the heart precursors. When fibronectin is completely absence, adherens junctions between the heart precursors are not well formed, suggesting that cell-matrix interactions are required for epithelial organization and that epithelial integrity is important for migration of myocardial progenitors [40]. The extracellular matrix Tenascin-C, is also associated with cell motility [25], [26]. Like Ccbe1, Tenascin-C is also expressed in the cardiogenic fields and disappear once the heart fields fuse [41]–[44]. However, no distinct phenothype was observed in *tenascin-C-null* mice [45]. Furthermore, Ccbe1 also has been associated to extracellular matrix remodelling and migration [40]. In zebrafish, Ccbe1 act as an extracellular matrix guidance molecule that regulates the budding and migration of lymphangioblasts from the anterior cardinal vein [27].

Interestingly, our results indicated that increased c*Ccbe1* levels resulted in expansion of Hnk1-expressing domain in both CNC cells and heart tube region, while loss of c*Ccbe1* decreased Hnk1 signal mostly in the heart tube region. Hnk1 plays a role in the migration of neural crest cells [25], [26], and is normally present in the cardiomyocyte precursor [41]–[44].

How ECM molecules guide the heart precursors in their migration is still unclear, however it has been proposed that they can bind and sequester signalling molecules, releasing them upon proteolytic maturation mediating their availability [46]. A recent report showed that CCBE1 affects lymphangiogenesis by enhancing the cleavage of VEGF-C by the metalloprotease ADAMTS3 [47]. It is known that the major regulators such as the, VEGF, Wnt, BMP, FGF and Nodal signalling pathways, can regulate both cell movements and fate of cardiac precursors [48]. For example, it was demonstrated that Wnt3a guides the movement of cardiac progenitors by a new mechanism involving RhoA-dependent chemorepulsion [49]. Furthermore, we cannot exclude that the integration and coordination of different signals from different pathways are important for proper cell migration guidance. Recently, it has been shown that the convergence between BMP and Wnt pathways regulate cardiac progenitors migration through Smad1 [50].

In addition, the migrating heart precursor cells also undergo a high proliferative process. Therefore, incorrect formation of the heart tube in cCcbe1 morphants can be also related with impaired cell proliferation. Indeed, it has been shown that alterations in the proliferation of cardiac precursor cells affect the migration of the precursors towards the midline [51]. Immunohistochemistry analysis of the proliferation marker PHH3 revealed that knockdown of c*Ccbe1* leads to decreased proliferation of the cardiac progenitor cells. These data indicate that in addition to the impaired migration, the absence of c*Ccbe1* also causes a defective cell proliferation of the cardiac progenitors. Interestingly, studies of proliferation performed in our lab, support these findings in which mouse embryonic fibroblast isolated from mCcbe1 KO show less proliferation when compared with the WT littermates.

In conclusion, here we address the role of c*Ccbe1* in early cardiogenesis. In chick, *cCcbe1* is expressed in both FHF and SHF progenitor populations, and that later becomes restricted to the SHF. This suggests that c*Ccbe1* is downregulated as the progenitor cells differentiate towards more definitive cardiac phenotypes. Upon c*Ccbe1*-loss-of-function during early cardiogenesis, the fusion of the two heart fields was incomplete or failed to close correctly leading to the formation of an aberrant heart tube. On the other hand, c*Ccbe1*-gain-of-function led to severe heart tube defects, including extreme non-midline *cardia bifida*. Furthermore, the levels of c*Ccbe1* influences Hnk1 expression and PHH3 positive cells in the cardiac regions suggesting a possible role in the migration and proliferation of the cardiac progenitors leading to an incorrect development of the heart. Taken together, these data support that c*Ccbe1* plays a role in early heart development and, therefore, is a candidate causative gene for cardiomyopathys. The relevance of Ccbe1 in mammalian cardiogenesis and the possible significance of Ccbe1 alterations in cardiac syndromes may deserve further studies.
